# The Willingness of Community Psychiatric Management Physicians to Preferentially Recommend Long-Acting Injections in Beijing

**DOI:** 10.3389/fpubh.2021.779563

**Published:** 2021-11-19

**Authors:** Lefan Jin, Yun Chen, Junli Zhu, Qingzhi Huang, Bin Li, Ying Xu, Rui Xi, Wei Lu

**Affiliations:** ^1^School of Public Health, Capital Medical University, Beijing, China; ^2^Research Center for Capital Health Management and Policy, Beijing, China; ^3^The National Clinical Research Center for Mental Disorders and Beijing Key Laboratory of Mental Disorders, Beijing Anding Hospital, Capital Medical University, Beijing, China; ^4^Advanced Innovation Center for Human Brain Protection, Capital Medical University, Beijing, China; ^5^Policy Research Office, Beijing Institute of Mental Health, Beijing, China

**Keywords:** severe mental disorder, long-acting injection, recommendation willingness, community psychiatric management physicians, free LAI policy

## Abstract

**Background:** Severe mental disorders (SMD) impose a heavy burden on individuals, society, and the country. Under the background of deinstitutionalization, more and more patients return to the community, and the community psychiatric management physicians (CPMP) play an essential role in this process. Long-acting injection (LAI) is an important way to improve compliance and reduce re-hospitalization. Some districts in Beijing have implemented the policy of free LAI. This article aims to find out the willingness of CPMP to preferentially recommend LAI and provide suggestions for follow-up promotion.

**Methods:** All CPMP in 16 districts of Beijing were surveyed. A self-made electronic questionnaire was used to investigate the willingness to recommend LAI in priority. Descriptive statistics, Chi-square test, and logistic regression were used to analyze the data.

**Results:** The willingness of CPMP to preferentially recommend LAI is up to 80%. Participants aged 40–49, female, with higher self-evaluation of psychiatric management knowledge, managing patients who have used LAI in the past, and working in communities with the free LAI policy have higher willingness to recommend LAI in priority.

**Conclusion:** CPMP in Beijing have a positive attitude toward LAI, and most of them have the willingness to recommend LAI to the patients in priority. The recommendation willingness is the basis of prescription decision-making. Therefore, the coverage of free LAI policy should be further expanded in the future to improve the recommendation willingness and thus improve the injection rate of LAI.

## Introduction

Low drug-taking rate, high recurrence rate are always difficulties in treating patients with Severe Mental Disorders (SMD) (1–3). The previous literature showed that 5 years after initial recovery, the cumulative first relapse rate was 81.9% (4). Data from the Clinical Antipsychotic Trials of Intervention Effectiveness study also showed that 74% of patients had discontinued their medication before 18 months owing to insufficient efficacy, intolerable side effects, or other reasons (5). The World Health Organization defined severe depression, bipolar disorder, schizophrenia, and other psychotic disorders as SMD (6). SMD often accompany weak guardianship, a high poverty rate, and easy-to-make accidents. It has become a major public health and social problem, affecting economic and social development (7–10). Data from the Chinese CDC showed that mental disorders ranked highest in the disease burden in China, accounting for about 20% of the total disease burden (11). It was estimated that mental illness would reduce Productivity in China by more than $900 million between 2012 and 2030 (12). By the end of 2018, there were 6 million registered patients with SMD in China, with a reported prevalence of 0.43% (13). What's worse, according to Shi et al., mental health resources were still insufficient to meet the demands of mental patients (14). Compared with developed countries, China's mental health resources were generally deficient. According to the latest Mental Health Atlas 2017, in high-income countries, the number of mental health workforce was 71.70 per one hundred thousand population, among which were 9.36 psychiatrists and 16.84 registered nurses per one hundred thousand population (15). However, even in China in 2019, the number of mental health workforce was 15.06 per one hundred thousand population, among which were 5.54 psychiatrists and 6.84 registered nurses per one hundred thousand population (16). Inadequate mental health resources also lead to a more pronounced revolving door effect: patients cycle back and forth through treatment, relapse, and readmission (17).

For a long time in the past, hospitalization was the mainstream treatment for SMD. In the 1960s, the United States launched the “Deinstitutionalization Campaign,” which advocated the reduction of large mental hospitals and the development of a community-based treatment mode (18). In 2004 China started the 686 Programme to integrate hospital and community services for patients with serious mental illness (19). In 2009, this service was included in the National Basic Public Health Services Project, which aimed to provide basic public health services for every citizen (20). Under the hospital-community integration mode, psychiatrists provided community psychiatric management physicians (CPMP) with technical guidance and emergency treatment of patients, instead of following patients in the community in person (20, 21). Regoli et al. pointed out that the “deinstitutionalization” created options for localized care, provided an opportunity for more family involvement, and reduced the social marginalization of patients caused by hospitalization (18). There are more and more patients returning to the community in China. According to the latest statistics, in 2018, the number of patients managed by the Chinese community was 5 688 164, accounting for 94.90% of the patients on file (13). These patients in the community enjoy follow-up rehabilitation services provided by the CPMP (21). Therefore, how to provide better services for mental patients in the community is becoming an important issue.

The long-acting injection (LAI) is a lyophilized powder that needs to be reconstituted with sterile water to form an injectable suspension without affecting the original molecule (22). Evidence also suggested that LAI not only had the clinical advantages of increasing treatment compliance, reducing the risk of recurrence and the rate of readmission but also could reduce the overall treatment cost, reduce the use of medical resources, and the burden on caregivers (23–25). According to a systematic review of economic evaluations by Achilla and McCrone, the majority of studies demonstrated that risperidone LAI, relative to oral drugs, was associated with cost savings and additional clinical benefits and was the dominant strategy in terms of cost-effectiveness (26). Altamura et al. concluded that the use of LAI antipsychotics might be particularly valuable in the community, especially in community mental health centers where psychosocial interventions and early rehabilitation services may be combined with pharmacotherapy (27). Despite all the benefits described above, in the United States, the prescription rate for LAI in the early 2000s was only about 19–30%, even among patients with a known history of non-adherence (28–30). For the rehabilitation of patients with SMD, the Chinese government has made some efforts to promote long-acting injections in recent years. In the National Health Commission of the People's Republic of China, LAI therapy was recommended for patients with poor treatment compliance, weak or no family monitoring ability, and risk of causing accidents (21). What's more, LAI was included in the Essential Medicine List and National Insurance Medicine List of China (31, 32). Even some local governments, such as Shanghai, Wuhan, and Yunfu, have issued policies to provide free long-acting injections for the above SMD (33). However, it was unexpected that the utilization rate of LAI in China was still <3% (34).

The previous studies showed that the reasons for the low utilization rate of the LAI were complex and the attitude of mental health professionals including clinicians and psychiatric nurses may be one of the most important barriers. Studies on the attitudes of mental health professionals and psychiatric nurses toward LAI suggested that there was wide variation in the attitude of the mental health professionals to the use of LAI and offering to patients. Heres et al. found that 65 and 71% of psychiatrists surveyed believed that first- and second-generation LAI were unsuitable for first-episode psychosis; 68% believed that neither first-generation nor second-generation LAI was an appropriate treatment option for a relapse of mental illness (35). On the contrary, Patel et al. found that 89 and 75% of surveyed physicians, respectively, believed that LAI was associated with better compliance and lower recurrence rates; 72% believed that the advantages of LAI outweighed the disadvantages (36). In fact, Perkins et al. found that physicians who truly believed that LAI improved compliance were far more likely to use LAI than those who were dubious (37). Though at present, the treatment plan of the patient is prescribed by the psychiatrist and the prescription power of CPMP is limited, as a bridge between psychiatrists and patients, they can play an important role in the effective treatment of mental illness. Schultz et al. concluded that family physicians were able to identify early signs of illness, made referrals to appropriate mental health professionals, helped patients and their families cope with the devastating effects of schizophrenia, and encouraged a multidisciplinary approach to all aspects of illness (38). Therefore, CPMP's attitude and willingness to recommend LAI also have a great influence on the final utilization rate of LAI. However, there is still a lack of relevant research on the views and recommendations of CPMP on LAI in China. Therefore, to explore the willingness of CPMP to recommend LAI in priority and its influencing factors, the following studies were carried out.

## Data and Methods

All data in this study were collected from all the active CPMP of 16 districts in Beijing, a total of 934 participants, through the questionnaire surveys, which were in the form of an electronic questionnaire. This survey lasted for about 2 months from the end of November 2020 to the end of January of the next year. All respondents were voluntary and written informed consent was obtained. To maintain confidentiality, names were not required on the questionnaires. Of the 16 districts in Beijing, 5 districts (Fangshan District, Chaoyang District, Tongzhou District, Huairou District, Fengtai District) had implemented the policy of free LAI before the survey, while the remaining 11 had not.

In this study, several steps were used to select items for the questionnaire. First of all, the questionnaire was designed by referring to the contents of the general demographic questionnaire and the factors that might affect CPMP's attitudes. Secondly, expert consultation included four professors from Mental Health and Health Economics and five CPMP from communities, who assisted in scrutinizing the appropriateness. Thirdly, a pre-survey was executed with a small sample to modify the instruments. The final questionnaire was divided into two parts. The first part is “Investigation on the basic information,” including CPMP's sociodemographic characteristics and work characteristics. Sociodemographic characteristics include the gender, age, education degree, and annual income of the CPMP. Work characteristics include working years of community psychiatric management, the number of patients managed, working full-time or part-time, self-evaluation of psychiatric management knowledge, whether patients managed have used LAI in the past, and whether the area implemented the policy of providing free LAI. The second part is “Investigation on service intention of LAI,” including whether the CPMP is willing to recommend the LAI in priority.

The study's dependent variable is whether the CPMP is willing to recommend the LAI to poor treatment compliance, weak or no monitoring, and high risk of causing accidents patients in priority. And the independent variables including 10 variables divided into 2 categories: sociodemographic characteristics and work characteristics. Heres et al. pointed out that older psychiatrists were more likely to offer LAI to their patients (35). And Kim et al. confirmed that more experienced physicians recommended LAI significantly more frequently than those with less experience (39). While Hamann et al. showed that psychiatrists' age, gender, and work experience did not affect their attitudes toward LAI and the likelihood of using LAI (40). Finally, based on extensive reading of relevant literature and combined with the actual situation in China, these 10 representative variables were determined and various parameters were set.

Descriptive statistics of the study variables are reported using frequencies and percentages. To examine the impact of policy implementation, sociodemographic characteristics, and work characteristics on the willingness to recommend LAI in priority, there are two steps in statistical analysis. The first step is to use the chi-square test to perform a univariate analysis of the basic characteristics and willingness to recommend LAI in priority. The second step is multivariate logistic regression. The model is divided into two categories: one is for the overall CPMP, and the other is grouped for regression analysis. As described above, CPMP are grouped according to whether their area has the policy of free LAI. To test whether the policy effects of each model are the same, in addition to the multiple logistic regression for the full sample, logistic regression is also performed on the samples of each model. The univariate analysis is a preliminary exploration of the association between the independent variable and the dependent variable, and the binary regression analysis aims to further exclude the influence of other confounding factors, to finally determine the correlation between the independent variable and the dependent variable. All statistical analysis is achieved through SPSS 26.0.

## Results

Overall, 934 CPMP in 16 districts in Beijing were investigated in our census study. We find that the willingness of CPMP to preferentially recommend LAI for patients with poor treatment compliance, weak supervision or no supervision and a high risk of accidents and accidents is up to 80%. Results of univariate and logistic regression analyses show participants aged 40–49, female, with higher self-evaluation of psychiatric management knowledge, managing patients who have used LAI in the past, and working in communities with the free LAI policy have higher willingness to recommend LAI in priority.

### CPMP Disposition and Sociodemographic Characteristics

The basic information of 934 investigated CPMP is shown in Tables 1, 2. Overall, 934 respondents returned their questionnaire (100.0%). Of the 934 CPMP surveyed, 788 (84.4%) prefer to recommend LAI therapy for patients with poor treatment compliance, weak supervision or no supervision, and a high risk of accidents and accidents. The majority of the CPMP are female (72.7%), are between 30 and 39 years old (41.6%), have a bachelor's degree or above (59.9%), and are primary CPMP (47.1%). Four hundred and sixteen CPMP (44.5%) have been working on community psychiatric management for 3 years or less. The majority of the CPMP manage <100 patients (54.5%), 539(57.7%) CPMP are working full-time. The annual income of 623 (66.7%) CPMP is no <70,000 yuan, that is, no less than the per capita income level of Beijing residents in 2020 (41). In terms of self-evaluation of the knowledge of prevention, 95.4% of the CPMP think that their knowledge reached the average level or above. 620 (66.4%) CPMP manage patients who have received the LAI in the past.

**Table 1:** Univariate analysis of factors influencing the willingness of CPMP to recommend LAI in priority: Sociodemographic characteristics.

**Characteristic**	**Assignment**	**Preferential recommendation for** **LAI (*N* = 934)**		**Total (*n*/%)**	**χ^2^**
		**Yes (*n*/%)**	**No (*n*/%)**		
Gender					0.701
Male	0	211 (82.7)	44 (17.3)	255 (27.3)	
Female	1	577 (85.0)	102 (15.0)	679 (72.7)	
Age (years)					17.881^***^
≤ 29	Assign a value of 1–4 from young to old	89 (76.1)	28 (23.9)	117 (12.5)	
30–39		317 (81.5)	72 (18.5)	389 (41.6)	
40–49		241 (91.0)	24 (9.0)	266 (28.5)	
≥50		140 (86.4)	22 (13.6)	162 (17.3)	
Education degree					0.256
Bachelor degree or above	Assign a value of 1–3 from low to high	469 (83.9)	90 (16.1)	559 (59.9)	
College degree		248 (85.2)	43 (14.8)	291 (31.2)	
Below college degree		71 (84.5)	13 (15.5)	84 (9.0)	
Annual income (yuan)					4.738^**^
<70,000	0	251 (80.7)	60 (19.3)	311 (33.3)	
≥70,000	1	537 (86.2)	86 (13.8)	623 (66.7)	

** *0.05,*

*** *0.01*.

**Table 2:** Univariate analysis of factors influencing the willingness of CPMP to recommend LAI in priority: Work characteristics.

**Characteristic**	**Assignment**	**Preferential recommendation for** **LAI (*N* = 934)**		**Total (*n*/%)**	**χ^2^**
		**Yes (*n*/%)**	**No (*n*/%)**		
Working years of community psychiatric management (years)					12.951^***^
0–3	Assign a value of 1–3 short to long	332 (79.8)	84 (20.2)	416 (44.5)	
4–7		199 (86.1)	32 (13.9)	231 (24.7)	
≥8		257 (89.5)	30 (10.5)	287 (30.7)	
Number of patients under management					9.951^***^
<100	0	412 (80.9)	97 (19.1)	509 (54.5)	
≥100	1	376 (88.5)	49 (11.5)	425 (45.5)	
Working status					57.700^***^
Full time	0	471 (87.4)	68 (12.6)	539 (57.7)	
Part-time	1	317 (80.3)	78 (19.7)	395 (42.3)	
Self-evaluation of psychiatric management knowledge					29.032^***^
Extremely familiar	Assign a value of 1–5 from not familiar to extremely familiar	58 (93.5)	4 (6.5)	62 (6.6)	
Very familiar		325 (89.8)	37 (10.2)	362 (38.8)	
Moderately familiar		372 (79.7)	95 (20.3)	467 (50.0)	
Slightly familiar		30 (83.3)	6 (16.7)	36 (3.9)	
Not at all familiar		3 (42.9)	4 (57.1)	7 (0.7)	
Whether patients managed had used long-acting injections in the past					39.416^***^
Yes	1	556 (89.7)	64 (10.3)	620 (66.4)	
No	0	232 (73.9)	82 (26.1)	314 (33.6)	
Whether the area implemented the policy of providing free long-acting injections					2.742^*^
Yes	1	277 (87.1)	41 (12.9)	318 (34.0)	
No	0	511 (83.0)	105 (17.0)	616 (66.0)	

* *0.10;*

*** *0.01*.

### Univariate Analysis Results

The univariate analysis affecting the willingness to recommend LAI in priority is shown in Tables 1, 2. In terms of social demography, univariate analysis shows there are statistically significant differences in the preference to recommend LAI among different age groups and annual income (*P* < 0.10). In terms of work characteristics, there are statistically significant differences in the willingness to recommend LAI preferentially among CPMP with different factors such as working years of community psychiatric management, the number of patients managed, full-time/part-time status, self-evaluation of psychiatric management knowledge, whether patients managed have used LAI in the past and whether the area implemented the free LAI policy (*P* < 0.10) (Table 2).

### Logistic Regression Analysis Results

Table 3 presents the results of logistic regression of the policy's impact on the willingness of CPMP to recommend LAI in priority. Regression results for all CPMP show that the 5 factors, the gender and the age of the CPMP, self-evaluation of psychiatric management knowledge, whether patients managed have used LAI in the past and whether the area implemented the free LAI policy have impacts on whether CPMP recommend the treatment of LAI in priority for patients with poor compliance, weak monitoring or no monitoring, and patients with a high risk of causing accidents (*P* < 0.10).

**Table 3:** Binary regression analysis of factors influencing the willingness of CPMP to recommend LAI preferentially when they come from areas with or without the free LAI policy.

**X**		**All CPMP (*N* = 934)**		**CPMP from the area with the free LAI policy (*N* = 318)**		**CPMP from the area without the free LAI policy (*N* = 616)**	
		***B***	***OR***	***B***	***OR***	***B***	***OR***
Gender		0.389^*^	1.476	0.427	1.532	0.350	1.420
Age	30–39	0.093	1.098	−0.127	0.881	0.200	1.221
	40–49	0.828^**^	2.288	0.689	1.991	0.896^**^	2.450
	≥50	0.173	1.189	−0.028	0.972	0.288	1.334
Working years	4–7	0.174	1.190	0.554	1.740	0.006	1.006
	≥8	0.260	1.296	0.659	1.934	0.079	1.082
Education degree	College degree	0.106	1.112	0.001	1.001	0.127	1.135
	Below college degree	0.088	1.092	0.233	1.262	−0.080	0.923
Annual income		0.230	1.259	0.092	1.097	0.310	1.363
Number of patients managed		0.193	1.213	0.254	1.289	0.161	1.175
Working status		−0.018	0.982	−0.022	0.978	−0.002	0.998
Self-evaluation of psychiatric knowledge		0.450^***^	1.569	0.306	1.358	0.498^***^	1.646
Patients managed had used LAI		0.987^***^	2.684	0.903^**^	2.466	1.044^***^	2.840
Policy situation		0.405^*^	1.499	–	–	–	–
Constant		−1.857	0.156	−0.829	0.436	−2.106	0.122
Hosmer-Lemeshow Tests (*P*-value)		0.100		0.597		0.193	

* *0.10;*

** *0.05;*

*** *0.01*.

Among the 934 CPMP, 318 are from the districts (Fangshan District, Chaoyang District, Tongzhou District, Huairou District, Fengtai District) that implemented the free LAI policy. Another 616 are from areas where the free LAI policy hasn't yet been implemented. Binary logistic regression analysis was used to explore the factors influencing the priority of the two groups of CPMP to recommend LAI to patients. Two regression results show that whether patients managed had used LAI in the past is the main influencing factor. And for 616 CPMP who came from the districts without the free LAI policy, the regression results show that the influencing factor is the CPMP's age and their self-evaluation of psychiatric management knowledge (*P* < 0.10).

## Discussion

LAI was shown to be more effective in preventing readmissions and improving medication compliance in patients with schizophrenia than oral drugs (42, 43). Nevertheless, LAI was seldom prescribed in the treatment of schizophrenia. For example, LAI was prescribed at 23.5% in France and 29% in the UK, which were already higher than anywhere else in Europe (44). Similarly, only about 13% of patients with schizophrenia spectrum disorders were treated with LAI in the US and <3% of patients in China were treated with LAI (34, 45). Since LAI was an effective way to treat SMD patients in the community and CPMP also played an important role in the community (27), this study was conducted among CPMP in 16 districts in Beijing. To explore the willingness of CPMP to recommend LAI in priority and found its influencing factors, our studies were carried out.

This study shows that for patients with poor treatment compliance, weak home monitoring ability or no monitoring, and with the risk of causing accidents, more than 80% of the CPMP prefer LAI for treatment, which is significantly higher than the existing research results. For example, in a survey of psychiatrists in northwest England, Patel et al. found that only 4% listed LAI as their first choice for long-term maintenance treatment for schizophrenia (36). There may be explained as follows: as mentioned above, in recent years, China's central government and some local governments have successively issued relevant policies to encourage the implementation of LAI for SMD patients, the advantages of LAI were gradually recognized by doctors in China; On the other hand, perhaps more importantly, in the process of treating patients with SMD, CPMP mainly play the role of “recommending drugs” and “managing patients,” which may be different from the attitude of psychiatrists who really have the right to prescribe LAI.

The results show that age and gender are two of the main factors affecting the willingness of CPMP to recommend LAI in priority. In this study, female CPMP are more likely to recommend LAI and accord with the result of West et al., which was a previous cross-sectional study of psychiatrists in the US and showed that non-white and female psychiatrists were more likely to prescribe LAI (30). This study finds that CPMP aged 40–49 are more likely to recommend LAI than those in other age groups. Heres et al. (35) also proved that age was an important influencing factor, but it concluded that older psychiatrists, especially those older than 50 years old, were more likely to offer depot formulation to their patients. However, the results were different from some other studies, such as (36, 40). Hamann et al. showed that psychiatrists' age and gender did not affect their attitudes toward LAI and the likelihood of using LAI (40). Patel et al. investigated attitudes of European physicians and found that gender was not significant neither in the univariate regression nor in the multivariate regression. As of the age, in the univariate regression analysis, the age of the CPMP was significantly associated with the proportion of patients willing to receive LAI antipsychotic medication, while in the multivariate regression model, age was no longer associated with the willingness to receive LAI (46). It may be due to the differential diagnosis and treatment backgrounds in different countries, and the younger and male CPMP in China may prefer the more prudent and commonly use oral drug therapy when recommending treatment. Among the demographic factors, this study also finds that work experience doesn't show a significant impact, which confirms the results of Hamann et al. (40).

Another finding of the study is that CPMP who rate themselves higher on their knowledge of psychiatric management are significantly more likely to recommend LAI. Although the LAI has been clinically used in the US and European countries for a long time, it is still a relatively new drug in the clinical treatment of psychiatry at present. CPMP who lack LAI knowledge tend to exaggerate the side effects based on perceptual knowledge and are more cautious in their willingness. In the qualitative study of Iyer et al., some psychiatrists interviewed highlighted their lack of knowledge as well as their lack of confidence in prescribing LAI (47). Another study reported that more than 50% of psychiatrists believed that LAI had more numerous and severe adverse effects than oral formulations (36). Waddell and Taylor proved that knowledge regarding the side effects of LAI correlated positively with psychiatrists' use of LAI (48). The study of Patel et al. also suggested that knowledge predicted a 20% variation in physicians' overall attitude toward LAI (36). Therefore, to further promote the LAI, it is necessary to strengthen the knowledge popularization of LAI among CPMP.

The significant findings of the present study also include that CPMP who have managed patients with LAI in the past are significantly more willing to recommend LAI therapy. The reason may be that in the process of managing such patients, CPMP's knowledge and experience about LAI have been increased, and they have become more confident to recommend LAI in the future. In fact, there have been some studies on the attitude of clinical psychiatrists to LAI in the past and reached similar conclusions, such as (39, 49). In a review, Spanish scholars Parellada and Bioque concluded that the main reason why LAI was not widely prescribed from the perspective of clinicians were limited knowledge and experience, personal attitudes and beliefs, and controversial aspects of scientific evidence (50). The policy implications of this finding are to encourage CPMP to use LAI and increase their experience with LAI, thus forming a positive cycle and further improving the use of LAI.

Finally, this study finds that the path of free LAI policy's influence on recommendation willingness is complicated. On the one hand, CPMP in the districts with free LAI policy have a higher willingness to recommend; on the other hand, there are differences in other factors affecting the willingness to recommend between the two groups of CPMP. In districts with the free LAI policy, the only influencing factor is the CPMP's self-evaluation of psychiatric management knowledge. However, in districts without the free LAI policy, in addition to self-evaluation of psychiatric management knowledge, the influencing factors also include age and experience in managing patients with LAI. It shows that free LAI policy not only directly affects the recommendation willingness of CPMP, but also plays a moderating role in the influence of other factors. Actually, although most studies showed that LAI was more cost-effective and had additional clinical benefits compared to oral drugs, LAI was more expensive than oral drugs as well (26, 51). What's more, Iyer et al., Parellada and Bioque believed that the main barrier to the use of LAI was a high cost (47, 50). Therefore, Lindenmayer et al. thought one possible solution was lowering costs of LAI and including them in insurers' approved medication panels (52). And French health care system recognized schizophrenia as a “long-term disorder” qualifying for full health insurance cover with 100% reimbursement of drug costs, that was why the cost of drugs was not taken into account in the decision to prescribe a depot antipsychotic by French psychiatrists, though the cost of LAI was a strong argument against their use (49). In India, by contrast, ~3-fifths (59%) of the psychiatrists reported that they underused LAI to a certain extent, with the most common reasons that deterred them from using LAI being the cost (55.45%), which was thought to be related to the fact that most of the cost of treatment was out of patients' own pockets (53). This variation can be explained by the particularities of each country's health care system and by differences in health policies. In China, most communities are sponsored by the government, and CPMP also have a special status similar to civil servants and have a high enthusiasm for the implementation of government policies including the free LAI policy. Now that the effects of the free LAI policy have been proven, it is necessary to expand the coverage of the free LAI policy.

The main contributions of this study are as follows: Firstly, different from most studies on psychiatrists' attitudes toward LAI and willingness to recommend LAI (35, 36), this study took CPMP as respondents to investigate their willingness to recommend LAI. With the development of deinstitutionalization, patients with mental disorders gradually return to the community, and the role of CPMP in the community health prevention system should not be underestimated. Secondly, compared with previous studies on LAI attitudes of psychiatrists in developed countries, such as The UK (36), Germany (40), and South Korea (39), this study provides empirical evidence under a different context. China is still a developing country with a large population and its economic development is at a low to medium level, and unlike other developing countries such as India (54), China's political system has its particularity, and so does its medical and health care system. Therefore, this study also enriches the research on CPMP's attitudes to LAI in developing countries. Finally, compared with previous studies, this study adopts a census method to obtain data, which can avoid the bias caused by sampling.

Of course, this study also has some limitations, which should be addressed by future research. First, this study divided CPMP into two groups according to whether there is a free LAI policy. In fact, in the five districts that have a free LAI policy, the release time of the policy is not consistent. At the time of the investigation, some districts' policies had been implemented for more than 2 years, while some districts had just issued policies. Taking into account the problem of sample size balance, this study did not make more detailed groupings. Second, this study is a simple cross-sectional design, which only analyses the recommendation willingness of CPMP and its influencing factors on the cross-section. If a cohort study is carried out, the time order of antecedent consequences can be better proved before and after the implementation of the policy in the same region. Third, this study doesn't include area-level data and patient-level data, such as area relapse rate, patient health outcomes, patient behaviors, and characteristics. In addition, this is a country-specific study rather than a cross-national one. The study would be more informative if it included area and patient-level factors and comparisons between countries. It is necessary to further investigate these factors in future studies.

## Conclusion

In this study, a cross-sectional design was adopted to conduct a census survey of all CPMP in 16 districts of Beijing and to systematically evaluate the group's attitude toward LAI treatment and preference for a recommendation. It has been proved that some areas in Beijing have begun to implement the free LAI policy, which to some extent has improved CPMP's willingness to recommend LAI. CPMP, as an important force in the prevention and treatment of mental health in the community, can more effectively help patients to recover and return to society by using LAI, which is worth promoting vigorously. Under the background of deinstitutionalization and economic globalization, strengthening CPMP training and promoting free LAI policy have maintained social security and stability, and improved the level of treatment methods for SMD in China to be in line with international standards.

## Data Availability Statement

The raw data supporting the conclusions of this article will be made available by the authors, without undue reservation.

## Author Contributions

JZ contributed to the conception and design of the study. LJ, YC, JZ, QH, BL, YX, RX, and WL organized the data collection. LJ performed the statistical analysis. LJ, YC, and JZ wrote sections of the manuscript. All authors contributed to the article and approved the submitted version.

## Funding

This research was supported by the National Natural Science Foundation of China (Grant Nos. 71573182 and 71974133) and the Beijing Municipal Natural Science Foundation (Grant No. 9192004).

## Conflict of Interest

The authors declare that the research was conducted in the absence of any commercial or financial relationships that could be construed as a potential conflict of interest.

## Publisher's Note

All claims expressed in this article are solely those of the authors and do not necessarily represent those of their affiliated organizations, or those of the publisher, the editors and the reviewers. Any product that may be evaluated in this article, or claim that may be made by its manufacturer, is not guaranteed or endorsed by the publisher.
